# Operationalising resilience for disaster medicine practitioners: capability development through training, simulation and reflection

**DOI:** 10.1007/s10111-019-00587-y

**Published:** 2019-09-12

**Authors:** Jonas Hermelin, Kristofer Bengtsson, Rogier Woltjer, Jiri Trnka, Mirko Thorstensson, Jenny Pettersson, Erik Prytz, Carl-Oscar Jonson

**Affiliations:** 1 Swedish Defence Research Agency (FOI), Stockholm, Sweden; 2 Department of Clinical and Experimental Medicine, Center for Disaster Medicine and Traumatology, Linköping University, Linköping, Sweden

**Keywords:** Resilience, Crisis management, Disaster medicine, Training programme, Cognitive skills

## Abstract

Resilience has in recent decades been introduced as a term describing a new perspective within the domains of disaster management and safety management. Several theoretical interpretations and definitions of the essence of resilience have been proposed, but less work has described how to operationalise resilience and implement the concept within organisations. This case study describes the implementation of a set of general resilience management guidelines for critical infrastructure within a Swedish Regional Medical Command and Control Team. The case study demonstrates how domain-independent guidelines can be contextualised and introduced at an operational level, through a comprehensive capability development programme. It also demonstrates how a set of conceptual and reflective tools consisting of educational, training and exercise sessions of increasing complexity and realism can be used to move from high-level guidelines to practice. The experience from the case study demonstrates the value of combining (1) developmental learning of practitioners’ cognitive skills through resilience-oriented reflection and interaction with dynamic complex open-ended problems; (2) contextualisation of generic guidelines as a basis for operational methodological support in the operational environment; and (3) the use of simulation-based training as part of a capability development programme with increasing complexity and realism across mixed educational, training and exercise sessions. As an actual example of a resilience implementation effort in a disaster medicine management organisation, the study contributes to the body of knowledge regarding how to implement the concept of resilience in operational practice.

## Introduction

The potential contribution of the diversely employed term *resilience* in, for example, the disaster management and safety management domains has been articulated by numerous researchers (Hills 2000; Comfort et al. 2001, 2010; Hollnagel et al. 2006, 2011; Manyena 2006; Woods 2015). This has resulted in various interpretations of resilience as, for example, the robustness of a system or the adaptive capacity of a system before, during, and after disturbances to find a new equilibrium (see Woods 2015). In addition, the question whether resilience actually is a useful concept has been raised (e.g. Klein et al. 2003; Manyena 2006). Although the literature on how to define resilience is abundant, the literature on how to recognise, operationalise and implement it for practitioners is not. To be made useful and contribute to the improvement of actual operations, it is necessary for the term resilience and resilience research to be associated more clearly with practitioner vocabulary and practices (see also Lay et al. 2015; Woltjer et al. 2015; Woltjer 2019). The central stance of this article is that the contextualisation and application of resilience research is needed to facilitate systematic local implementation in crisis management. These implementations are regarded here as vital lessons to be shared to enable subsequent learning, and to make the body of knowledge regarding what practically constitutes resilience available to facilitate a wider systemic implementation in crisis management and critical infrastructure.

To increase the resilience of critical infrastructure organisations, Branlat et al. (2017) and Herrera et al. (2019) described a set of high-level and domain-independent guidelines, which are derived from the resilience literature. These guidelines are called the DARWIN Resilience Management Guidelines (DRMG; DARWIN 2019) and are suggested to be used by crisis management practitioners on various policy, management, and operational levels at EU, national, regional, and organisational scales (Herrera et al. 2019). The DRMG contain suggestions of both activities and sets of triggering questions aimed at aiding organisations in analysing how they can improve their resilience and develop practitioner-directed training packages.

The purpose of this case study was to demonstrate how an educational, training and exercise programme aimed to enhance organisational resilience in crisis management can be designed and executed. The case study describes how a subset of the DRMG was contextualised and introduced at an operational level, i.e., to a Swedish regional medical crisis management team, through the development and implementation of a capability development programme. This case study, thus, constitutes a contribution to applied research within the Resilience Engineering and Crisis Management domains as a demonstration of an effort to provide design and development processes, strategies, and capabilities to accomplish resilient performance in complex socio-technical systems (see, e.g. Wreathall 2006). This case study, thus, contributes to narrowing the gap between resilience in theory and resilience in practice.

## Background

This section briefly introduces key aspects of resilience and training in crisis management. To conclude the section, the use of simulations in exercises and training is addressed, which is a key enabler for training for resilience.

### Operationalising resilience

Many definitions and concepts of resilience have been suggested in research areas such as Resilience Engineering, Community Resilience, and Disaster Resilience (Holling 1996; Hills 2000; Comfort et al. 2001, 2010; Hollnagel et al. 2006, 2011; Manyena 2006; Longstaff et al. 2010; Woods 2015). In these areas, the definitions of resilience commonly connote a need for safety–critical organisations to develop their adaptive capacity and prepare for the unexpected in a pro-active manner. Moreover, the potential for adversity challenges the resilience of organisations by demanding the ability to gracefully extend their performance and add and sustain adaptability (Woods 2015, 2018). However, published definitions of resilience may be perceived as rather abstract from a practitioner perspective. The DRMG described by Herrera et al. (2019) are proposed as a stepping stone of operationalising the theoretical concept of resilience, to make it more accessible for organisations aiming to increase their resilience.

As resilience is a broad concept, the DRMG divide resilience into different capabilities, which are each described in what is called a capability card (DARWIN 2019; Herrera et al. 2019). The thirteen capability cards focus on a specific aspect of resilience and cover what is relevant for the aspect (Table 1). The capability cards are proposed to be used to guide policymakers, managers, and practitioners in (further) enhancing the resilience of crisis management teams, organisations, and systems as a whole. The suggestions in the capability cards are stated generically, as the DRMG are designed to be applicable to critical infrastructure independent of domain, geography or organisation.

**Table 1:** The thirteen capability cards in the DRMG (DARWIN 2019)

Theme	Capability card
Supporting coordination and synchronisation of distributed operations	Promoting common ground for cross-organisational collaboration in crisis management
	Establishing networks for promoting inter-organisational collaboration in the management of crises
	Sharing information on roles and responsibilities among different organisations
Managing adaptive capacity	Enhancing the capacity to adapt to both expected and unexpected events
	Establishing conditions for adapting plans and procedures during crises and other events that challenge normal plans and procedures
	Managing available resources effectively to handle unusual and changing demands
Assessing resilience	Assessing community resilience to understand and develop its capacity to manage crises
	Identifying sources of resilience: learning from what goes well
	Noticing brittleness
Developing and revising procedures and checklists	Systematic management of policies
Involving the public in Resilience Management	Communication strategies for interacting with the public
	Increasing the public’s involvement in Resilience Management
	Supporting Development and Maintenance of Alternative Working Methods

Each capability card includes a description of a certain aspect of resilience and suggests suitable interventions and actions to be implemented or considered by policymakers, managers and practitioners at various levels. All capability cards follow the same structure, where the suggested activities are complemented with a set of triggering questions to be used to guide self-reflection within organisations. As an example, the capability card Identifying sources of resilience: learning from what goes well suggests activities that focus on understanding work-as-done (Hollnagel 2017) both in daily operations and during crises through methods such as interviews and workshops. The capability card also suggests analysing how and when procedures are applied to understand which existing practices work well. The suggested triggering questions cover themes such as adaptive capacity, operational margins, resources, monitoring, goal trade-offs, and dependencies and interactions (e.g. Hollnagel 2017; Woods 2006). Examples of such triggering questions are: Which strategies (e.g. working methods or contingency procedures) can be used to handle a sudden loss of capacity and/or increase in demands? Which margins are available in everyday operational situations that can be used to handle suddenly increased demands? Which monitoring mechanisms are put in place by the organisation to anticipate and assess possible threats that may occur in the future? How are formal and informal networks nurtured that are useful in handling crises?

Additionally, the concept cards list examples of actual practises from organisations within healthcare and air traffic management that illustrate the aspect of resilience.

### Learning resilience by training for the unexpected

The operational level of crisis management is by definition a complex environment with high levels of uncertainties, and rare events (Sinclair et al. 2012), which are both dynamic and beyond what can be managed through standard operating procedures (Ford and Schmidt 2000). Although crises are generally expected in complex domains, the specifics and temporal occurrence of a crisis are often a surprise event. These events can be further divided into a situational surprise (that can be fitted into the current understanding), and a fundamental surprise (that challenges basic assumptions of the situation) (Lanir 1986). Due to the complexity of crises and their inherent aspects of surprise, crisis managers often face situations where it is impossible to determine a single most correct solution to the problems encountered. These situations, or problems, can be described as open problems, as opposed to closed problems, where it is possible to define a correct solution in advance (Fredholm 1988). Managing open problems is not just about finding a certain solution, but also about the need to identify and define the problem to be solved (Ellström 1992). This analogy corresponds to the assessment of a situation and how crisis management personnel identify and define an unfolding crisis situation. This process relies on the personnel’s cognitive skills to observe, understand, and assess the situation they face (Klein and Baxter 2009).

Cognitive skills are dependent on mental models, which are the set of causal beliefs of how things fit together and are used to predict the effect of an action or decision (Klein and Baxter 2009). Developing these cognitive skills is generally not a question about just acquiring more knowledge, but rather restructuring existing mental models (Klein and Baxter 2009). During restructuring, previous beliefs can be changed or rejected to better suit gained insights based on new experiences. This sense-making process (see also Klein et al. 2007; Rankin et al. 2016; Weick et al. 2005) demands that the learners discover for themselves how their mental models must be developed (Klein and Baxter 2009). This is closely related to the individual’s reflection and critical questioning of how work is conducted (Ellström 2004).

Reflection can be seen as a conscious effort by a learner to examine their own mental models. Dewey (1910) described reflection as “active, persistent, and careful consideration of any belief or supposed form of knowledge in the light of the grounds that support it, and the further conclusions to which it tends” (p. 6). Dewey’s (1910) and Schön’s (1987) analyses of reflection invite the idea of training reflection skills regarding resilience to better cope with complexity, uncertainty, and the unexpected. Connecting to Woods’ (2015, 2018) notion of resilience, being able to reflect on how and when current strategies need to be adjusted, adapted, extended or sustained, seems critical for resilient practice. Furthermore, considering the dynamics and hands-on nature of acting in a crisis, Schön’s notions of reflection-on-action, (reflecting before and after a crisis management activity), and reflection-in-action (reflecting during the crisis management activity) both seem valuable for programmes aimed at increased resilience. Moreover, structured evaluation of exercises using after-action review (AAR) methods (Rankin et al. 1995; Morrison and Meliza 1999; Jenvald 1999) supports trainees in discussing and consolidating new mental models, which is one of the ultimate goals of a training event.

As noted by Mann et al. (2007), guidance for educators to develop reflective ability is scarce, although learning is one of the central outcomes of reflection. In a healthcare context, a review of reflection development studies by Mann et al. (2007) suggested that reflection may be stimulated through certain academic (traditional, classroom) interventions. When preparing for the unexpected, however, traditional ways of training with a focus on lectures and training on instructions are less suitable (Schaafstal et al. 2001). In addition, many traditional training settings focus on a sequential and controlled development. The controlled nature of such designs could hinder trainees to fully develop their skills (Ford and Schmidt 2000). Klein (2011) also cautioned against training with an overly analytical approach to problem-solving in complex environments. He pointed to the importance of using both an analysis path and an insight path in interpretations of complex situations. The insight path is a rapid experience-based interpretation tactic relying on expertise to identify associations and patterns, in comparison to the slow, but more systematic analysis path. Gaining experience is, thus, important to extend trainees’ abilities to imagine and recognise essential aspects of the situation. Requisite imagination (Adamski and Westrum 2003), or being able to creatively anticipate what could happen in an actual event, is an essential skill for successful coping with irregular and unexampled events. Scenario elements in training should, therefore, be representative of actual events. However, at the same time, the degree of predictability may vary to stimulate the participants’ thinking about expected, unexpected, and unexampled events (see Westrum 2006). Learning by experience would aim to enhance the capability to anticipate the courses of events in future actual work.

Combined, these aspects indicate a need for an active learning environment that is not necessarily fully controlled and sequential, but instead open to innovative ways of tackling open problems. For crisis management, more advanced action-oriented training programmes that iteratively employ reflection before, during, and after various crises could be argued to be useful for increasing resilience. The approach employed in the current study combined *education*, *training* and *exercises* in a process of successively increased complexity and access to relevant feedback to facilitate developmental learning. This aimed to stimulate experiential learning and reflection-in-action as well as reflection-on-action, using and building on practitioners’ expertise in relation to the concept of resilience.

### Simulations for learning experiences

Simulation-based training (SBT) is a widely used training method and one of the foundational uses of simulation (Banks 2010). It has been shown to have the potential for effective training with good transfer effects to the corresponding real situations (see, e.g. Maran and Glavin 2003; Wolfe and Crookall 1998). The benefits of SBT include controlled, safe, and affordable training environments that can be tailored to specific educational objectives and learner backgrounds, where both task-oriented as well as cognitive skills can be repeatedly trained and explored (Salas et al. 2008). This training typically follows an experiential learning paradigm, where skills and knowledge are built based on experience of performing and doing (Kolb 1984; Maran and Glavin 2003; Wolfe and Crookall 1998).

The use of simulations in training settings ranges from simple pen and paper table-top exercises (TTX) to full-scale exercises featuring real resources. In training experienced disaster management personnel for complex crisis situations, a sufficient level of realism, or fidelity, is needed. While a lower level of fidelity can be beneficial for novice participants, the training of experts may require a high level of fidelity, as they can process the complexity and use it in their decision-making process (Alessi 1988). Woltjer et al. (2006) suggested using realistic scenarios in training sessions and exercises to bolster organisational resilience. They used such simulations to study resilience within organisations challenged by demanding situations where time, resources, and experience were limited and not in parity with the demands of the situation at hand. Elaborate and realistic simulations offer rich situation descriptions and cues to gain experience and train perceptual discrimination skills, i.e. the ability to make distinctions between previously indistinguishable concepts or events (Klein and Baxter 2009). Exposing trainees with an already high proficiency level to realistic and complex simulated environments may, thus, build experience and enhance recognition-primed decision-making (see Klein 2008), learner self-efficacy (Jonson et al. 2017), and requisite imagination (see Adamski and Westrum 2003).

Another well-known benefit of using simulation-based training is the ability to provide immediate feedback. Feedback is widely accepted as a necessary learning component of simulation-based training (see, e.g. Fanning and Gaba 2007; Moreno 2004). Given a sufficient level of fidelity, SBT offers the possibility for learners to see the effect of their decisions and actions, thus giving them feedback on their understanding of the situation and the fitness of their mental models (Klein and Baxter 2009). Encouraging reflection is of particular importance for successful education and training of crisis management personnel in terms of open problems and resilience. In SBT, this can be achieved through the ability to generate dynamic developments of events. Typically, SBT is also considered a cost-efficient way of learning lessons before they are experienced in actual operations. This is of great importance for a competent crisis management organisation, especially as it is often comprised of several individual actors that need to work together. Exercises create possibilities of lessons learned, which is what drives development and evolution (Crichton et al. 2009).

## Method: contextualisation of resilience guidelines

The capability cards that are part of the DRMG are based on resilience literature and are all formulated as generic guidelines independent of domain and organisation. While they do contain examples from healthcare, a structured implementation requires an adaptation effort where the guidelines are contextualised through adjustment and association to organisation-specific policies and practices, as well as educational and training interventions that consider the processes and practices in the organisation. Due to the wide scope of the DRMG, the current study focused on implementing a subset of these guidelines that was considered especially relevant for the targeted participants in the Swedish Regional Medical Command and Control Team (RMCCT). In the case of major incidents, the RMCCT takes responsibility and mandates over the healthcare resources in the region of Östergötland, Sweden.

The implementation of the DRMG began by a contextualisation process where the DRMG were placed into a national, domain-specific, and operational context. The contextualisation was done together with experts that either work with policymakers in advisory roles or work themselves as policymakers for Swedish crisis management. One part of the contextualisation was to describe resilience as the adaptive capacity of critical societal infrastructure operations that allows them to retain adequate functionality and continuity in case of societal disruptions. This description is intended to be in line with the Swedish national guidance on the common use of terminology in crisis management (MSB 2017). Furthermore, a major part of the contextualisation of the subset of capability cards was to describe the content of these through the notions of basic capabilities and prerequisites. The basic capabilities were formulated to clarify what the RMCCT need to achieve to be able to be resilient, while the prerequisites described what they need to do to achieve the capabilities in an operational context.

**Fig. 1:**
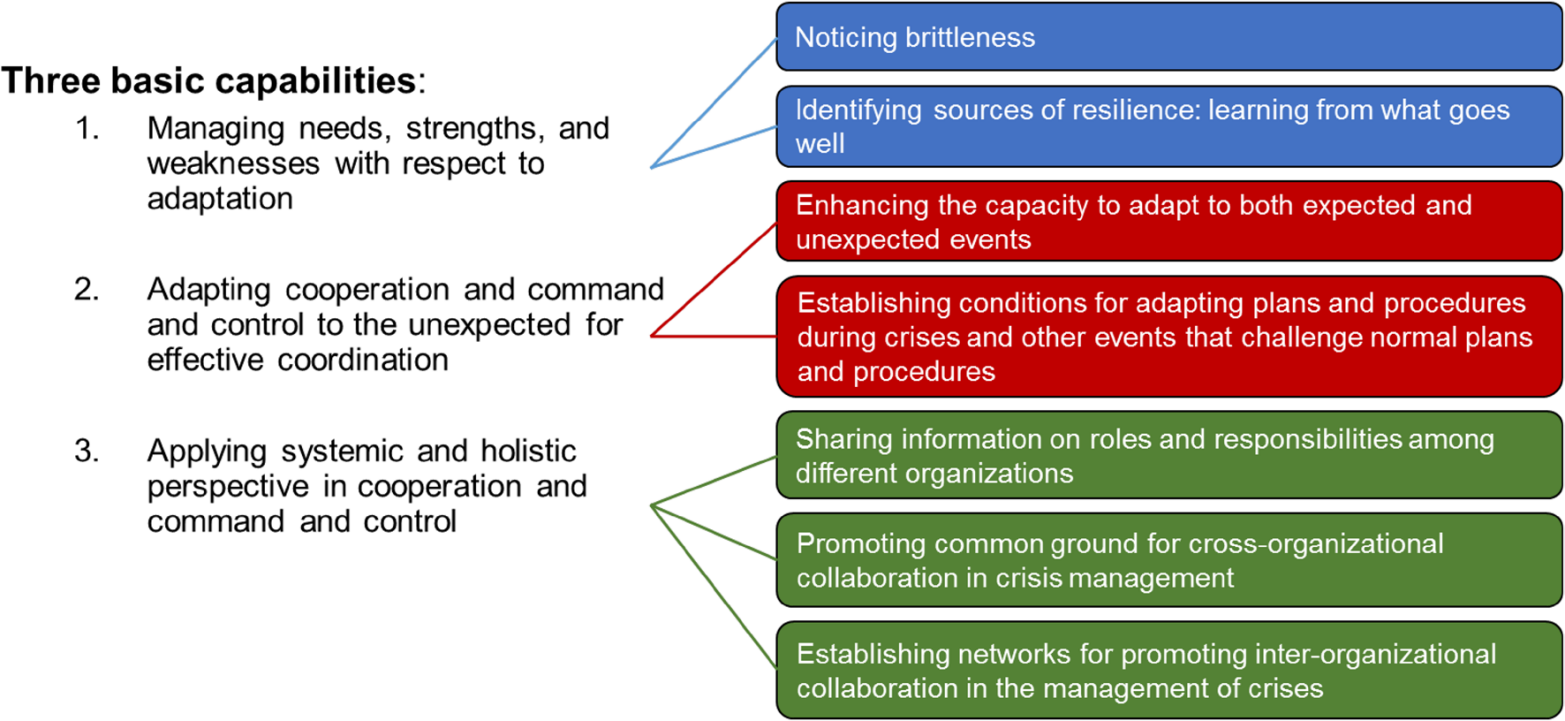
Relation between basic capabilities and a subset of the DRMG capability cards

The capabilities were based on concepts described by the subset of capability cards. The use of domain-familiar terminology (such as capabilities) makes it easier for the participants to understand the central concept of resilience, and brings the general concept of resilience closer to the actual operations conducted by the RMCCT. The capabilities and their connection to the capability cards are shown in Fig. 1.

To provide the participants with more specific support in an operational environment, the capabilities were complemented with six prerequisites that facilitate the before-mentioned capabilities and resilient performance. The list of prerequisites was given to the participants as an easy-to-remember methodological support, applicable in an operational environment. The purpose of the prerequisites was to provide teachable, supportive and actionable resilience guidance, aligned with existing terminology used by the RMCCT. The prerequisites were formulated as:

- *Managing goal conflicts within and between actors.* Crisis management includes several actors that have different goals and prioritisations with their response, in part due to their different roles of responsibility or their assessment of the situation. This can be explored through questions like: How do we identify, avoid or manage goal conflicts and prioritisation? What is our capability to prioritise and compromise between different goals during an ongoing response?
- *Revealing vulnerabilities.* A challenge that crisis management actors face when trying to obtain an overview of a complex crisis and manage surprise events is to identify potential vulnerabilities or minor events that risk evolving into major disruptions, e.g. situations where the response contains several new dependencies of other actors. This is related to triggering questions like: How do we identify vulnerabilities and manage them during a response? What vulnerabilities are there in our organisation and our capabilities? How could these affect our response?
- *Understanding crucial assumptions.* The assessment and management of a crisis is founded on several explicit and inexplicit assumptions in crisis management. These will influence how events or actions taken by other actors are assessed and anticipated. These assumptions can be explored through triggering questions such as: What assumptions do we have regarding common praxis? How do we identify invalid assumptions?
- *Being aware of constraints.* All actors act according to certain constraints regarding, e.g. resources, competence and mandates. These constraints can be discussed through questions such as: within what margins are we able to manage a crisis, and what happens when we are close to or cross these boundaries? What can we do to extend our capabilities and overcome certain constrains?
- *Having systematic and capability understanding.* Crisis management can include several different actors with different capabilities interconnected through a network of relationships and dependencies. This understanding can be facilitated through discussions regarding: What actors are included in the crisis management, and what are their preconditions and capabilities? How can these actors form an understanding of the potential or actual collaborations?
- *Using success factors.* Actors can find it challenging to identify and apply experiences that have been successful during previous events. This can be facilitated through asking: How do we identify and document successful actions? How do we determine success factors and use them during future events?

As with the capabilities, the prerequisites were also based on the content in the capability cards in the DRMG (Table 2).

**Table 2:**
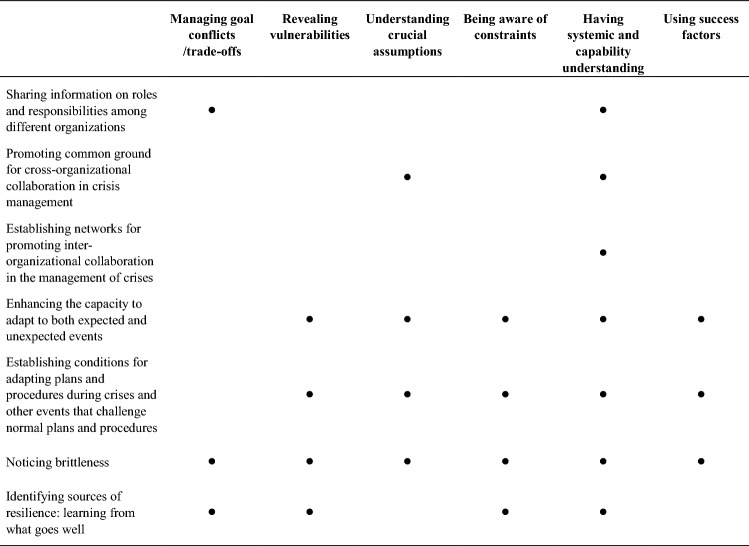
Overview of the relationship between prerequisites (horizontally) and capability cards (vertically)

The contextualisation also included an adaptation of an existing paper-based self-reflection tool that identifies sources of brittleness and resilience in preparation of a crisis. The tool, called Analysis Aid (Johansson et al. 2018), uses three perspectives to support reasoning of the organisational maturity regarding resilience: (1) a process-oriented perspective, (2) a capability perspective, and (3) a staffing perspective. The tool was adapted by integrating triggering questions from the DRMG into these perspectives, as well as including self-reflection on the six prerequisites. Throughout the programme, the participants were asked to use Analysis Aid as a basis for formalised reflection-on-action.

## Results: capability development programme and evaluation

This section reports how the capability development programme was designed and how it was perceived and evaluated by the participants.

The case study focused on the Regional Medical Command and Control Team (RMCCT) in the region of Östergötland, Sweden. Five practitioners from this team were the main participants in the case study. The DRMG were applied through a capability development programme combining education, training and exercise between May and October 2017. The activities were designed to be suitable for the designated organisation and their current practices and level of resilience, and consisting of three types of activities:

1. Educational activities through lectures that introduced the main concepts of resilience and the DRMG, adjusted to the RMCCT work. The focus was to increase the knowledge and awareness of these concepts among the participants, as a starting point for discussion and reflection-on-action.
2. Training activities through workshops, where the theoretical concepts were applied on different scenarios or evaluated by the practitioners. The focus was to facilitate discussions and reflection-on-action on how the concepts fit in an operational situation.
3. Exercise activity through a Table-Top Exercise (TTX) and a Command Post Exercise (CPX), where the concepts could be evaluated in an operational setting. The focus was to allow the practitioners to operationalise the DRMG concepts to gain experience of using them during operational work and stimulate reflection-in-action using the concepts.

**Fig. 2:**
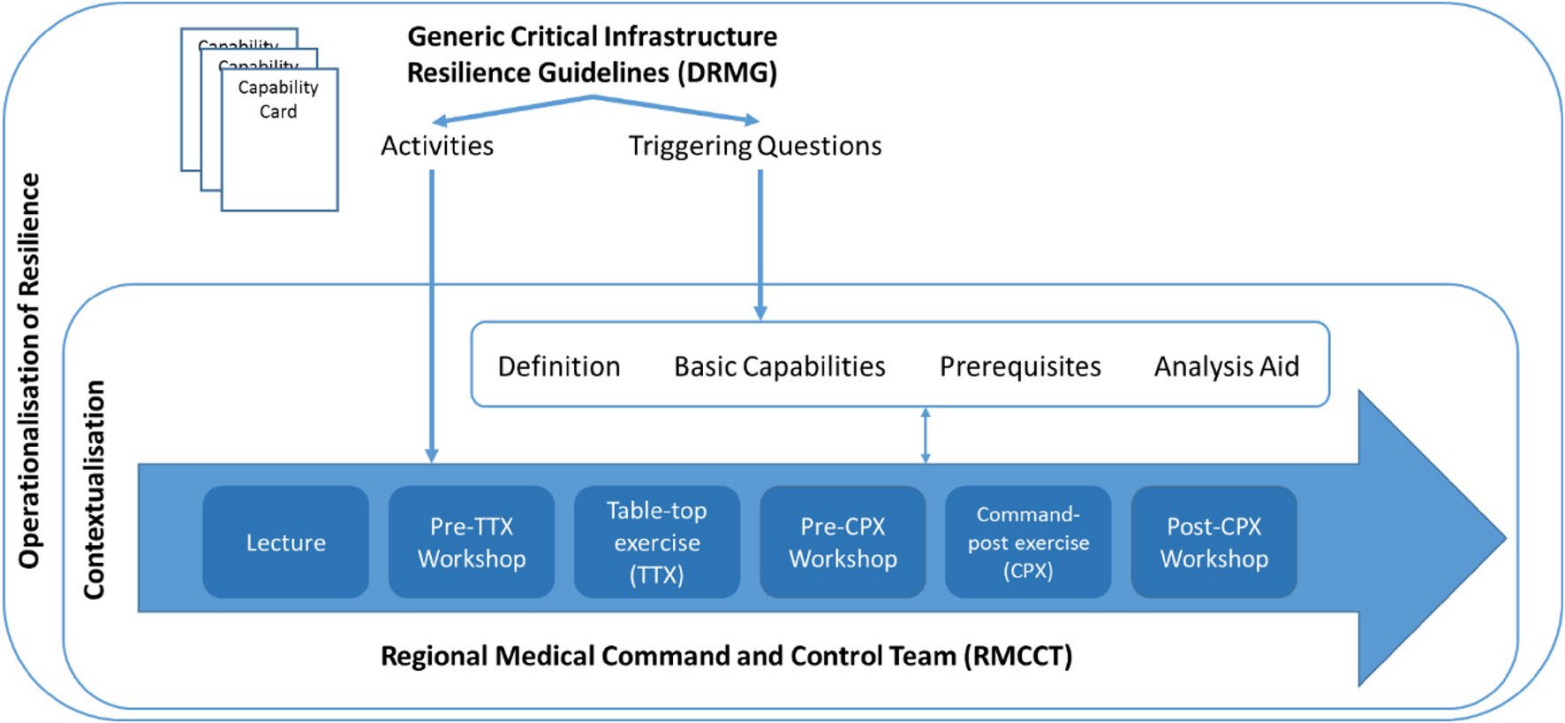
Overview of the sessions that were part of the capability development programme (blue arrow), with the means by which Resilience was contextualised (resilience definition, basic capabilities, prerequisites, and Analysis Aid). The activities and triggering questions are described in the DRMG capability cards

The activities were performed during six sessions (Fig. 2). During these sessions, one or two types of activities were performed (e.g. educational and training activities).

The purpose of the chronological order of the sessions was to gradually familiarise the participants with a resilience-based practice and to address any questions regarding resilience as a starting point. The approach enabled the participants to discuss, reflect, and adjust their understanding of the content to fit into their operational procedures in activities with increasing complexity and fidelity. The final large-scale (CPX) exercise allowed the participants to recognise and enact the resilience-based practice in combination with their common procedures to further reinforce learning.

### Description of the capability development programme

The implemented capability development programme consisted of a series of six sessions aimed at the same group of participants from the RMCCT, mixing educational, training, and exercise activities. The content of each session was planned to successively facilitate reflection and feedback loops to the participants on how to increase the level of resilient thinking in their crisis management.

*Session 1* consisted of an educational activity in the form of a lecture. The objective was to introduce the target audience to the purpose and content of the capability development programme. The lecture also gave the participants a basic understanding of resilience with respect to the DRMG and an outline of how the DRMG relate to current praxis and policies of the regional medical management. In this respect, the lecture was an important foundation for the following sessions. The content of the lecture was carefully formulated to be relevant and understandable for the operational personnel in the specific domain, as well as the national and regional context. Thus, only a small part of the lecture dealt with resilience in generic terms. Instead, it was framed by the contextualised initial description of basic capabilities and prerequisites. Definitions of resilience were only briefly discussed to contrast the academic and practitioner perspectives. The need for adaptation of ways of working in the face of dynamically changing circumstances was a central theme. The capabilities and prerequisites were presented as tools to enable adaptation to current situations in a more structured and predictable manner. While adaptation is necessary, the lecture contained a discussion on how adaptations can have the desired effects, but that there is also a risk of unanticipated, unintended or undesired consequences. The aim of using the prerequisites as tools is to reduce this risk and increase desirable effects. During the lecture, the prerequisites and how these resonated with their experience in operational work were discussed in detail with the participants. The day concluded with the participants discussing and reflecting on commonalities between resilience, and current practices and guidelines, as well as the difference of applying the concept of resilience at different levels, such as the national and the regional team task levels.

Aspects from all the prerequisites were recognised by the participants and were discussed in relation to earlier experiences. Some examples are presented here. The importance of understanding the purpose of different organisations and managing goal conflicts ahead of incidents was noted, as lack of time makes this harder to do during actual crises. To be able to manage these conflicts, the actors need to have a pragmatic approach during collaboration. Identifying erroneous situation understanding is an example of revealing vulnerability. An erroneous understanding was said to be worse than an incomplete understanding, as a lack of information naturally prompts the search for more information. The participants exemplified the need for understanding crucial assumption as being sceptical to initial information reports during major incidents as they often contain mistakes and misunderstandings. A common incorrect assumption among other actors is that patients will be transported to the nearest medical facility, which is not necessarily the case. The more indistinct hierarchy of decision rights in the healthcare management, compared to the police, is an identified constraint, which can lead to a lower degree of adherence to decisions. Collaboration between organisations is important, but it is also time consuming. Knowing when to collaborate in an efficient manner demands a proper system understanding. Success factors were already included in the RMCCT working methodology, and therefore something that they identify and document.

*Session 2* consisted of a workshop where the RMCCT participants discussed three scenarios, chosen to represent a broad variance of events. The scenarios were (1) a chemical spill in a city centre, (2) an influenza pandemic affecting personnel and the local community, and (3) a cyberattack and IT-outage of the regional healthcare systems. For each scenario, the participants were asked to describe what actions they would perform in a similar event and why. Throughout the workshop, triggering questions were used to challenge the participants and facilitate their reflection and analysis of the scenario from a resilience perspective. Issues that were discussed included: risk of a lack of resources and requesting extra resources externally, situation understanding by and interaction between multiple stakeholders, logistics issues, communication challenges, uncertainties in the scenario, need for expertise, management tactics, duration of consequences, bottlenecks, adaptation needs, goal conflicts, and identification and monitoring of vulnerabilities. The workshop scenarios and some example discussion subjects are presented below.

Scenario 1. Carbon disulfide (CS_2_) is leaking from an industry located centrally in a city of 140,000 inhabitants. The leakage can continue for an additional 10–12 h and there is a risk that the gas will spread through the sewer system. The gas is volatile with a high risk of explosion. Spontaneous ignitions are occurring, resulting in release of sulphur dioxide (SO_2_).

The participants discussed not only how evacuation would be a primary goal in this scenario and how other resources (e.g. police and rescue services) would be used, but also how to prioritise between evacuation and stopping the leakage. Resources would be severely limited, until the arrival of ambulances from other counties. At that point, the problem could become the opposite with too many resources challenging the ability to maintain control.

Scenario 2. It is the third week of a phase 5 pandemic of the A/H5N1 virus. The capacity of the healthcare system is reduced as the personnel are also directly or indirectly affected by the virus. It is still unclear if and when current medical countermeasures will be effective.

In this scenario, the RMCCT would be supplemented by specific competence in disease control. Communication to the public would be important and needing to be coordinated on a national level. There would be a high risk of disinformation spreading through social media that would need to be monitored. Given that this scenario is so clearly in the healthcare domain, the risk of less collaboration with other actors would increase. Even if the healthcare system would manage the acute situation, there would be repercussions for a significant amount of time afterwards. It is a vulnerability that the fear of being infected might negatively affect the morale and effectiveness of the healthcare personnel.

Scenario 3. An extensive cyberattack has affected several of the healthcare IT-systems, such as X-ray, telephones and patient journal systems. Lacking the normal emergency response IT-system, all prehospital management is conducted using only mobile phones and the TETRA radio system. The TETRA system will run with some limitations to ensure capacity. The primary healthcare centres and hospitals are receiving large numbers of unannounced patients.

The discussion focused on different fallback routines, how medical equipment might be affected and how to redistribute patients to other counties and countries. When there are technical failures, the competence and skills of individual members of medical personnel are important for resilience. This raises the question whether the most experienced personnel should be on site at the medical facilities or if they should work at the crisis management level.

The final activity of the workshop consisted of the participants using the Analysis Aid to individually analyse and reflect on a fourth scenario—a generic shipping accident event. The Analysis Aid further stimulated reflection on subjects such as interactions with external roles, working methods, and information technology.

*Session 3* consisted of a Table-Top Exercise (TTX) using the methodology developed by Sandström et al. (2014). The main objective was to provide the participants with a first initial opportunity to apply and discuss the contextualised DRMG on a specific scenario in more depth. In addition to the RMCCT participants, the TTX had other participants from the regional medical service (e.g. pre-hospital, hospital and regional level), The Swedish Maritime Administration, the Swedish Police and regional rescue services. The scenario used during the TTX was centred on a shipping accident on the east coast of Sweden, involving a passenger ferry with 2000 passengers. The scenario was played out using the first five (out of six) phases described by Sandström et al. (2014), i.e. (1) pre-incident, (2) incident and alarm, (3) early phase, (4) acute phase and (5) late phase. The last phase, aftermath, was not used in this TTX. For each of the phases, the participants were asked to discuss what actions they would take and reflect on the interaction with other actors and the scenario, using the resilience prerequisites. The scenario included three different what-if cases during the fourth and fifth phases. The different cases were (1) that the ship was able to seek emergency port, (2) that it was unable to move, and (3) that there was an immediate risk of sinking. The focus of the TTX on high-level aspects and different possible what-if outcomes of shipping accidents aimed at preparing the participants for the CPX, without revealing the details of that specific scenario.

The TTX included discussions on various actors’ roles and functions, how to reach various roles and experts, mandates and decision rights, locations of resources, interactions, and information flow between organisations, commonalities to related actual events in the past, goal conflicts and trade-offs, working methods of interacting organisations, timing of cooperation between organisations, and use of commonly used technology. The actors, thus, gained an insight into the other organisations and were given an opportunity to discuss the collaboration and identify potential conflicts of interests. In general, increasing the understanding of each other’s organisations, goals, and ways of working in connection to the scenarios was one of the main recurring themes during the discussions, which was also reported by multiple participants as a useful outcome. For example, the TTX was helpful for clarifying in what way it was unclear who, how or when some actors (who seldom collaborate) would initiate collaboration. Moreover, discussions between various actors enabled reflection on assumptions regarding available medical resources in rescue helicopters and the possibilities to transport medical personnel onboard a ship in distress. One of the discussions that participants found useful was on potential differences in perspective between actors, and the implications of whether the event was regarded primarily as a mass casualty situation that happened onboard a ship, or primarily a maritime accident that included mass casualty.

*Session 4* consisted of a workshop to prepare the participants for the exercise to be conducted in session 5. The Pre-CPX workshop began with a lecture where the contextualised theoretical part of resilience (basic capabilities and prerequisites) was reintroduced to allow the participants to further incorporate the concepts, together with the experience from the previous sessions. Following the lecture, the TTX was discussed to gather lessons learned. With regard to the prerequisites applied to the TTX, the participants noted that it was useful to think about one’s own understanding of other’s goals. They reflected on differences of perspective between the participants regarding what it meant for an operation to be successful, on goal conflicts, on differences in assumptions, vulnerabilities, and the usefulness of pre-established plans with and early notification of other organisations in managing the goals of the operation. One of the participants noted that it was essential to have such an opportunity (i.e. a TTX) for reflection on the six prerequisites and apply the prerequisites concretely in a scenario. The discussions indicated that the TTX had provided new insights into the management of maritime accidents and raised several questions regarding the collaboration between actors. After the discussion, the participants used the Analysis Aid to analyse the TTX scenario. The aim of the workshop was to facilitate the participants’ reflection on how the resilience concept would be applied in actual crisis management through the operationalisation of the DRMG, with respect to the experience drawn from the TTX and previous discussions. This discussion triggered an exploration of issues such as detection of threats, triggering conditions for alarms, time scales of decision-making, task descriptions, coordination with other organisations, communication capabilities and technology, strengthening of resources, reporting understanding of the situation, information management and limitations in information flow and timing, teamwork, and indicators for monitoring the situation. The scale of the scenario and the large number of actors involved initiated reflections on how to achieve communication and shared understanding, as well as on how the current technical systems would cope.

The session was concluded by a detailed discussion of what would happen and what decisions would be taken during the first 30 min of a crisis, starting from the first notification of a collision between a cruise liner and an oil tanker. This information was used as the starting point for the scenario used in session 5.

*Session 5* consisted of an exercise with the objective to give the RMCCT the possibility to apply the contextualised DRMG during a crisis response and establish an actual experience of being in a resilience-demanding situation. The session had the format of a Command Post Exercise (CPX). The CPX utilised real groups, real tasks, actual ecological settings and social systems (see Drabek and Haas 1967), meaning that the scenario was executed in real time (duration of 5 h), that actual workplaces and information and communication systems were used (e.g. telephone and communication radio), and that real-life collaborative settings (e.g. representative personnel) were in place. During the CPX, the RMCCT consisted of the five participants that were part of the capability development programme, supplemented with additional personnel (e.g. functions such as PR and crisis support). The degree of fidelity of the CPX and the realism of the feedback the RMCCT would receive on their decisions during the scenario was considered to be key to facilitate a sufficient learning and evaluation environment. To create a sufficiently realistic setting, the CPX was based on the combination of both the computer-based and analogue simulation tools. To create realistic spatial and temporal feedback to the participants, a computer-based simulation tool (Forsgren et al. 2011) was used to keep track of all units and resources, such as ambulances, helicopters and the movement of patients. Simulation of the treatment and condition of individual patients was carried out using an evidence-based analogue simulation system (Rybing et al. 2016). The exercise used an indirect simulation approach for the RMCCT, which meant that they had no direct interaction with either simulation tool. Instead, the RMCCT acted and received information through their ordinary communication channels (e.g. telephone and communication radio). The implementation of decisions and actions taken by the RMCCT was carried out by different response cells (e.g. air- and seaborne operations, land-based operations, hospitals). In total, the response cells consisted of 15 different functions, each manned by subject-matter experts acting as the environment interacting with the RMCCT as well as the interface between the RMCCT and the virtual environment.

The scenario in the CPX was a large-scale maritime rescue operation requiring an extensive medical response operation. The scenario in the CPX consisted of a collision between two ships in calm weather off the east coast of Sweden at 08:25 on September the 5th, 2017. The ships involved in the accident were a large cruise ship and a medium-sized tanker. At the time of the collision, the cruise ship carried approximately 1700 passengers with a crew of 400. The collision caused relatively large damage to the cruise ship, requiring the ship to seek port immediately. The tanker sustained no personal injuries and only minor material damage. After the collision, both ships stayed afloat and capable of sailing under their own steam. The tanker moved south and exited the scenario, while the cruise ship moved north at 09:30. At 11:30, the cruise ship stopped for an hour due to suspected fire before continuing. At around 13:40, a full evacuation of the cruise ship was initiated. All remaining passengers were relocated to another passenger ferry to be transported to the port of Norrköping, with the ETA 15:30.

In total, 305 people needed medical evacuation from the cruise ship. The scenario was specifically designed to put additional strain on the command and control function by forcing a dispersal of resources to cover all eventualities resulting from vessel movements. Such a scenario design is not common in the exercises of the public health care services, which mostly include a specific incident site with one ambulance loading area. The standard operating procedure (SOP) within medical crisis management assumes the presence of a specific accident site located at a fixed geographical area, which is also accessible to the on-scene commander for the pre-hospital services. The scenario with a moving ship violated both assumptions. This forced a re-evaluation of the organisation of the medical resources onshore. It also demanded a high level of coordination with other organisations (e.g. Swedish Maritime Administration, Swedish Coast Guard, and the Swedish Joint Rescue Coordination Centre as on-scene commander). The maritime scenario is also considered challenging because it demands an organisation for casualty and medical transports from the vessel to shore and then further on to the hospitals. A remote incident site as in this scenario means that neither the ambulance incident commander nor the medical incident commander will have access to or obtain a full view of the incident site as they are used to. This increases the challenges in sensemaking as well as in logistical planning.

An example of the prerequisite of understanding crucial assumptions can be found when the cruise ship changed destination to a different port than the one previously assumed by the team, which is then communicated by the regional medical incident commander to the ambulance incident commander. This new information challenges crucial assumptions, in turn challenging both commanders’ strategies (where to send patients) and resource allocation (of ambulances), preparing for the expected surge of patients in proximity to the cruise ship’s new destination. An example of systematic understanding can be seen when the regional medical incident officer and medical transport officer decided to redistribute helicopters to hospitals in counties further away to facilitate long-distance transport with the aim to avoid local optimization of local transports and increase an overall time gain for medical transports.

Yet another example, recognising the constraints of limited number of medical staff and amount of equipment onboard, the RMCCT planned to use the specially trained personnel onboard the medical helicopter to support the emergency triage and transportation decisions on the cruise ship. The identification and use of previous success factors were exemplified during the CPX, when the RMCCT decided to use strategic distribution points to pool ambulance resources for later dispatch, when specific casualty clearing points could be defined and decided. The communication and collaboration between the Joint Rescue Co-ordination Center (JRCC) and the RMCCT were identified as a vulnerability, making the RMCCT request closer collaboration with the JRCC. The request was motivated by the consequences of not including the healthcare perspective from the RMCCT early on in the decision-making. To improve communication and collaboration, a healthcare liaison officer was located at the JRCC.

*Session 6* consisted of an after-action review (AAR) and activities to gather reflections on the CPX and the programme as a whole. The AAR was conducted as a focus group interview with the participants from RMCCT, led by a moderator. During the AAR, the course of events from the scenario was replayed and visualised using a dedicated tool (Andersson et al. 2008; Andersson 2009). The actions taken by the participants were discussed in the context in which they occurred. The scenario was divided into several phases separated by certain key events. Each phase was discussed regarding how the response was organised, what was considered challenging and difficult, as well as the application of the six prerequisites. In this way, all participants were given the opportunity to reflect on their own and others’ actions. For some of the events in the exercise that had been particularly troublesome, the exercise directors explained their decisions and the motives for pushing the events in a specific direction.

The discussions during the after-action review covered aspects and insights regarding communication with other agencies, goal conflicts, cross-organisational understanding and combined sea-, land-, and airborne resource management. The participants claimed that they took an active resilient perspective during the CPX regarding how to establish communication with other agencies and what to communicate with them. The scenario was described as complex and dynamic, as unforeseen events happened, and the participants reported that they had difficulty understanding the scale of the scenario. Proactive decisions to ensure collaboration with the JRCC were taken, but due to misunderstandings this did not facilitate the communication as intended until late in the scenario. The appointment of a liaison officer was discussed during this session, weighing arguments on whether they would have taken that decision during an actual event or not. The participants also discussed their respective understanding of work processes in and mandates of other agencies, and the risk of presuming understanding of each other’s organisations. Resource management strategies were discussed and the participants noted some of the lessons learned regarding the importance of strategic distribution points of resources with respect to the time delays between land-based and sea-based resources. The participants reported that the tempo of the CPX was beneficial for learning, but that the team was too understaffed to be able to analyse the situation and coordinate the work sufficiently. One participant reported that most of the time during the exercise was spent on the telephone and that the amount of information received was overwhelming, leaving little time for analysis. During the exercise, the RMCCT had no possibilities to request additional personnel to the staff. While noting that a lack of resources is always a risk, they would normally be supplemented with additional personnel. In this case, it was rather the opposite as one of the key participants of the RMCCT had to make an unplanned leave in the middle of the exercise. While the prerequisites were used during the reoccurring staff meetings, the chief of staff reported that the intention had been to allow the staff to prepare reflections on the prerequisites 15 min before each meeting. However, due to the high workload of the individual staff members, it was not possible to make these formalised preparations. The issue with an understaffed RMCCT was noted as a vulnerability during the CPX. The participants noted that the concept of vulnerabilities is good, but they also raised the issue that a single major unsolvable vulnerability (e.g. understaffing) risk overshadowing other, avoidable, vulnerabilities.

After the AAR, the participants were asked to conduct a self-reflection on the scenario and actions taken during the CPX once again, using the Analysis Aid. In the concluding discussion, many of the participants expressed the value of such an AAR and that it helped them to learn from what had happened. An important statement by one of the participants was that it was useful to have several workshops in combination with a TTX to grasp the concepts and how to apply them. The pre-CPX workshop was highlighted as vital for the understanding before the application in the CPX. The analysis aid was also pointed out by one participant as a useful tool.

### Evaluation questionnaires

A subset of the results of collected data from the questionnaires that were distributed to the participants in conjunction with the sessions is reported in Tables 3 and 4. For each item in the questionnaire, the participants answered through an ordinal scale of five steps, ranging from Fully disagree to Fully agree. Questionnaires were used after each session to be able to adjust the contents of the subsequent activities in accordance with the participants’ feedback. The questionnaire supplied after the CPX included several more detailed questions on how the exercise improved different aspects of their understanding and capability regarding Swedish crisis management.

**Table 3:**
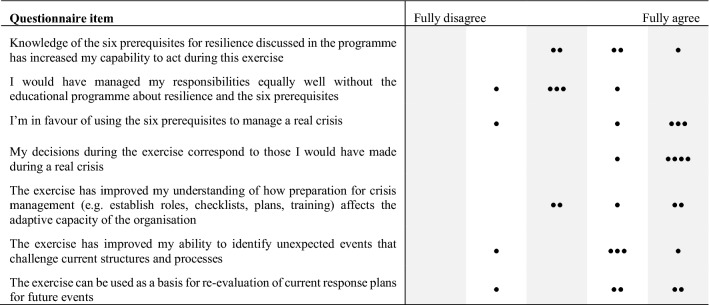
Results from the questionnaires answered after the CPX (*n* = 5)

**Table 4:**
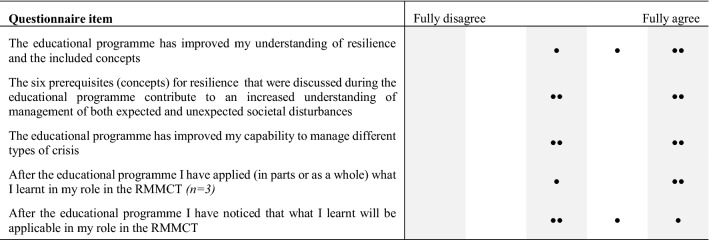
Results from the questionnaires answered 6 months after the last session in the programme (*n* = 4)

The reported results in the questionnaires should primarily be viewed as an indication of the participants’ attitudes towards the different parts of the programme. This study was, however, not a formal experiment as it was not possible to use a control group due to the inherent differences in the RMCCTs, which make them difficult to compare. Moreover, the complexity and dynamics of these types of crisis management scenarios typically make strict comparison across exercises and teams very difficult, if not impossible. Overall, this makes the ceteris paribus (all other factors remaining equal) condition of formal experimentation impossible to uphold. This, together with the fact that there were relatively few participants and that an ordinal scale was used in the questionnaires, no further statistical analysis would be meaningful in this case. Further analysis of all questions is outside the scope of this article, and therefore not presented here.

## Discussion

The case study reported in this paper focuses on how to educate, train and exercise personnel involved in command and control functions of crisis management and crisis response, to contribute to their resilience. The case study used a combination of educational, training, and exercise activities to inform operational personnel about resilience, and guide them to reflect on this concept and how to apply it in their operational environment. The format of the training programme with lectures, workshops, table top exercises and a concluding command post exercise was perceived as useful by a large entity of the participants. In a majority of the aspects, the overall impressions of the participants were positive, based on enquiries made in conjunction with the different programme activities. The workshops generated overall positive remarks with regard to the design of the activities, the level of increased understanding of the term resilience and the included concepts.

In general, the evaluation questionnaire answers illustrate the participants’ positive attitudes and experience of the CPX and the capability development programme as a whole. Overall, during the programme, the involved participants expressed positive feedback on the relevance and effect of the programme. Notably, after an actual major incident a few months after the programme, a former participant credited the resilience programme as contributing to maintaining control and managing the incident. We argue that the evaluations presented here are reasonably convincing of a benefit to the studied RMCCT, suggesting that other crisis management organisations should explore how their teams could benefit from the DRMG and try interventions similar to this capability development programme. While it is difficult to conclusively evaluate the DRMG directly in this paper, the implementation and contextualisation of the DRMG in this case study were deemed as successful, which invites for further research on applying the DRMG and programme to other RMCCTs and crisis organisations. In this respect, the DRMG are now being used as a basis for future courses provided by the Center for Disaster Medicine and Traumatology (KMC) in the region of Östergötland, Sweden.

### Contextualisation of high level guidelines to operational level

The basis of the resilience programme was the European level Darwin Resilience Management Guidelines (DRMG). The guidelines themselves were written primarily to be used as guidance for policymakers when drafting national or domain-specific guidelines, training programmes or procedures. The implementation used in this case study did not prescribe a certain procedure for the participants to follow. Instead, the focus of the intervention was to educate and train the participants in a new way to describe how they approach complex and unknown situations. The participants were supplied with a set of operational methodological support tools consisting of a list of six aspects, called prerequisites, to be used during operations that highlight certain critical cues in the situation. The actual operationalisation of the resilience concepts and these contextualised prerequisites were left to their expertise discretion. In this sense, the programme used a co-creation process of resilience behaviour. This is in line with Klein and Baxter’s (2009) suggestion to develop practitioners’ perceptual discrimination. By introducing a new framework through the resilience programme, the practitioners were able to tune into a new set of cues in situations, thus learning to recognise aspects that they previously could not.

With respect to the critique by Klein (2011), stating that an overemphasis on an analytical approach hinders the use of tacit expert knowledge, care was taken not to frame the methodological support tools, such as checklists. Instead, the combination of sessions and methodological support aimed to challenge the participants’ conceptual understanding of the world (i.e. change their mental models). Through discussion, triggering questions and the prerequisites, the aim was to improve their ability to observe critical cues (i.e. increase their perceptual discrimination). As such, it can be advocated that this case study tried to support situation interpretation both through insight and analysis, and thus avoid the negative aspects pointed out by Klein (2011). In addition, it was important to challenge the participants’ assumptions of the situations, other actors and so forth, with the aim of making them more attentive and pre-emptive towards unexpected situations and chains of events.

A key factor in the current case study was the level of experience of the involved participants. All participants had several years of experience of crisis management in their domain, and several worked as medical professionals or instructors in crisis management in parallel with their command and control function. This background was likely instrumental in the successful joint contextualisation and operationalisation of the resilience concept shared by researchers and practitioners, as experienced and instructing personnel are likely to have used more self-reflection in their work, besides being good at what they do and having experienced many different situations and exercises.

### The use of self-reflection

In the evaluations, the participants mentioned the importance of self-reflection when addressing resilience matters. One of the participants expressed that it was only during the fourth session (Pre-CPX Workshop) that it dawned on him and he obtained a sense of what resilience actually means. The importance of tools, such as the list of prerequisites, was pointed out in this aspect as well. Self-reflection can be argued to be a key component in the development and maturity of newly acquired knowledge (Borodzicz and van Haperen 2002). Some participants acknowledged this by stating in the questionnaires that the pre-exercise workshops were helpful in reflecting on the meaning and implementation of the new concepts, whereas others stated that most lessons were learnt during the concluding exercise. This might indicate that the duration and appropriate time for moments of reflection during a training programme vary between individuals. This provides support for the many-session capability development programme employed in this study, providing ample and organised opportunity for reflection throughout. The result of reflection mostly paying off in the final exercise is in line with Klein and Baxter (2009), as it was only during the high-fidelity exercise that the participants had an opportunity to actually test their understanding and identify needs to adjust their mental models. While time for reflection and discussion is important, so is also the possibility to evaluate conclusions drawn by reflection through enactment in a sufficiently realistic setting.

Supporting Schön’s (1987) notions of both *reflection*-*on*-*action* and *reflection*-*in*-*action* seems useful for enabling increased resilience through guided self-reflection. The sessions encouraged reflection, both after actual events that the participants had experienced in their career and before (anticipating) future hypothetical events not yet encountered. Reflection was also actively encouraged during multiple sessions before, during, and after a large command post exercise. Thus, both reflection on-action and reflection in-action were implemented interchangeably and iteratively. The implemented educational programme may serve as guidance for educators and trainers to develop reflective ability in crisis management.

### The use of simulations in training

Virtual environments and simulations are useful for training complex problem-solving and situation assessments (Klein and Baxter 2009). This case study used simulations with increasing complexity and fidelity during the resilience training programme. A Table-Top Exercise (TTX) with a low level of fidelity was used to highlight several weaknesses in the processes for joint collaboration between the land-based and the air-/sea-based organisations. During the TTX, the participants had a chance to discuss cross-organisational issues and learn that their counterpart reflect differently facing a common scenario. Such sessions can be valuable to identify both shared goals and goal conflicts between organisations, identify non-defined communication protocols or division of decision rights between actors. The TTX also served as an important preparation not only for the more high-fidelity CPX, giving important input to the CPX planning and preparation, but also for the participants to identify problems beforehand that would otherwise be a hindrance during the CPX.

While the use of simpler paper-and-pen simulations such as TTX is important, the use of more advanced simulation tools in the training and assessment of command and control functions can provide realistic and dynamic scenarios. These realistic scenarios enable exercises with open problems, which enhance the resilience aspects of the conducted operations, thereby reaching further than more traditional ways of managing and conducting such exercises (Woltjer et al. 2006). The level of realism in the different representations of in CPX is strongly connected to the focus of the specific exercise, which in this case was on embedding the RMCCT in a context where external actors had a major part and the decisions made by the team had realistic effects on the scenario, through representative consequences and time delays. Often, these types of exercises focus on the internal procedures in a command post and collaboration within the staff, allowing the representation of the surrounding environment to have lower fidelity. However, in this study, applying more realism in external parameters in the exercise was the focus of the interaction between the RMCCT and other actors. If a scenario with a lower degree of complexity and a simulation with a lower degree of fidelity and realism had been used, interdependencies with other actors would not have been possible to address. Some response cells were manned by single individuals and the level of workload on these functions were underestimated, making them overly strained during the real-time scenario. This affected particularly the ambulance dispatch function. Consequently, the response time for ambulance dispatch was prolonged, which unintentionally increased the challenge for the RMCCT to manage the situation.

One part that particularly contributed to the case study was the use of a dynamic and event-based scenario in the CPX. The dynamics were mainly constituted by allowing the vessel in distress to move along the coastline. Event-based actions following previously made decisions forced the participants to plan and adapt accordingly. This increased the awareness of, for example, brittleness in the organisation and showed the importance of robustness with regard to communications and interactions with other actors as well as a sustained adaptability. The scenario was specifically designed to force the participants into a situation that the current SOP was not suitable for. This scenario, thus, worked as a “baffling event” (Klein and Baxter 2009), challenging the way the participants initially approached and tried to solve the situation, which revealed needs to adjust their mental models. During the evaluation, the participants reported an appreciation of the way that the exercise demonstrated how a lack in adaptability might hamper operations in the face of unexpected scenarios and events. In line with this, one participant suggested that resilience and the resilience concepts should “… be applied more often in exercises in order to reach a volume of numbers of events”. This would suggest a desire for more opportunities to train on managing unexpected events and increase the cognitive skills connected with this.

The use of simulation tools was part of the success of the capability development programme, but it should also be noted that this was due to the effective integration and use of these tools in conjunction with a defined education and training programme (Oser et al. 1999). Solitary use of simulation tools does not suffice. Instead, these needs to be an integrated part of the training and should also be designed and calibrated to meet the desired effects of the training (Salas et al. 2008). In this case study, by implementing the capability development process, a natural upscaling of fidelity and realism was utilised for increasing the degree of problem-solving and need for mental models managing resilience. The resulting increased degree of complexity made it easier for the trainees to adapt the DRMG, develop their mental models and understand the need for resilience thinking.

## Conclusions

The aim of this article is to describe a resilience training programme that was applied in a Swedish regional medical command and control team. The article shows an example of how resilience concepts can be introduced to, contextualised for, and operationalised with operational crisis management personnel, through the combination of education, training and exercise. The case study shows that guidelines developed for generic critical infrastructure policymakers at different organisational levels can be successfully implemented when the guidelines are flexible enough to allow for contextualisation, giving organisations some leeway on the specific implementations.

The actual operationalisation of the DRMG was performed by the participants themselves with guidance supplied through the programme sessions. While the contextualisation of the DRMG provided them with a domain specific resilience programme and operational methodological support, the specific implementation of the concepts was based on their expertise and judgement. The researchers and practitioners managed to gradually fuse the knowledge and training in resilience with current practices, as observed throughout the resilience programme, eventually leading to the programme influencing the team’s practices and own training programmes.

This article documents how a series of workshops, lectures, and exercises aggregated into a coherent training programme can facilitate researchers and practitioners to jointly operationalise and contextualise resilience. Throughout the programme, the practitioners conveyed that they were able to associate the various aspects of their work context to a broad range of resilience subjects in a relevant and meaningful way, such as resources management, situation understanding, interaction between multiple stakeholders, uncertainty management, need for expertise, management tactics, adaptation needs, goal conflicts and trade-offs, working methods of interacting organisations, timing of coordinated activities, and identifying and monitoring vulnerabilities.

The wealth and relevance of resilience subjects addressed, participant engagement, and feedback collected from the various sessions indicate that the practitioners benefited from the programme and were able to apply the concepts introduced, which was the purpose of the programme. The reception of the approach is, therefore, considered successful. It may be regarded as an implementation of the design of a resilience capability development programme through cognitive transformation (see Klein and Baxter 2009), and multiple diverse opportunities for reflection on-action (before and after simulated and real events), and in-action (during simulated crisis management operations) (see Schön 1987).

In the case study, the use of training activities with cross-organisational representation was shown to be important, not only as a way to increase cross-organisational knowledge, but also as an opportunity to challenge the participants in their perception of other organisations and thereby test their mental models of how collaboration should take place. The activities, such as the TTX, were regarded to be useful to support the definition of lines of communication and identification of roles and capabilities. Implementing learning resilience in a medical command and control team by adapting a capability development process in the form of a capability development programme with increased use of simulation, complexity, fidelity and realism in consecutive steps was one success factor for this study. Combining educational interventions with exercises, particularly a table-top exercise (TTX) and a computer-assisted command post exercise (CPX), with a high level of realism was a key element for several participants in understanding the concept of the DRMG and the need for mental models of resilience in the operational environment of medical response in crisis management situations.

While this case study only covers a single implementation of a resilience capability development programme, the programme constitutes an example of an actual implementation of *resilience in practice* within the domain of disaster medicine management.
